# Randomized trial of nutrition education added to internet-based information and exercise at the work place for weight loss in a racially diverse population of overweight women

**DOI:** 10.1038/nutd.2013.39

**Published:** 2013-12-23

**Authors:** A Carnie, J Lin, B Aicher, B Leon, A B Courville, N G Sebring, J de Jesus, D M DellaValle, B D Fitzpatrick, G Zalos, T M Powell-Wiley, K Y Chen, R O Cannon

**Affiliations:** 1 Cardiovascular and Pulmonary Branch, National Heart, Lung and Blood Institute, National Institutes of Health, Bethesda, MD, USA; 2 Nutrition Department, Clinical Center, National Institutes of Health, Bethesda, MD, USA; 3 Diabetes, Endocrinology and Obesity Branch, National Institute of Diabetes and Digestive and Kidney Diseases, National Institutes of Health, Bethesda, MD, USA

**Keywords:** obesity, adiposity, weight loss program, racial disparity, women's health

## Abstract

**Objective:**

Obesity in the United States is highly prevalent, approaching 60% for black women. We investigated whether nutrition education sessions at the work place added to internet-based wellness information and exercise resources would facilitate weight and fat mass loss in a racially diverse population of overweight female employees.

**Methods:**

A total of 199 (average body mass index 33.9±6.3 kg m^−2^) nondiabetic women (57% black) at our institution were randomized to a 6-month program of either internet-based wellness information (WI) combined with dietitian-led nutrition education group sessions (GS) weekly for 3 months and then monthly with shift in emphasis to weight loss maintenance (*n*=99) or to WI alone (*n*=100). All were given access to exercise rooms convenient to their work site. Fat mass was measured by dual-energy X-ray absorptiometry.

**Results:**

WI+GS subjects lost more weight than WI subjects at 3 months (−2.2±2.8 vs −1.0±3.0 kg, *P*>0.001). Weight (−2.7±3.9 vs −2.0±3.9 kg) and fat mass (−2.2±3.1 vs −1.7±3.7 kg) loss at 6 months was significant for WI+GS and WI groups (both *P*<0.001), but without significant difference between groups (both *P*>0.10); 27% of the WI+GS group achieved ⩾5% loss of initial weight as did 18% of the WI group (*P*=0.180). Blacks and whites similarly completed the study (67 vs 74%, *P*=0.303), lost weight (−1.8±3.4 vs −3.3±5.2 kg, *P*=0.255) and fat mass (−1.6±2.7 vs −2.5±4.3 kg, *P*=0.532), and achieved ⩾5% loss of initial weight (21 vs 32%, *P*=0.189), irrespective of group assignment.

**Conclusion:**

Overweight women provided with internet-based wellness information and exercise resources at the work site lost weight and fat mass, with similar achievement by black and white women. Additional weight loss benefit of nutrition education sessions, apparent at 3 months, was lost by 6 months and may require special emphasis on subjects who fail to achieve weight loss goals to show continued value.

## Introduction

In the United States, nearly 70% of adults are overweight and more than 30% of women are obese.^1^ The prevalence of obesity is particularly high among underrepresented minority populations, ∼60% for black women.^1^ Previous studies have indicated that employees in the United States spend large portions of the work day sedentary, which likely contributes to energy intake in excess of energy expenditure.^2, 3^ Because of the adverse impact of physical inactivity and obesity on health care costs, many organizations have initiated ‘wellness' programs, which may include facilities at the work site to encourage exercise.^4, 5, 6, 7, 8, 9, 10, 11, 12, 13, 14, 15, 16, 17, 18^ Although these programs have often resulted in improved fitness for participants, weight loss has been more difficult to achieve.^5, 6, 13, 14, 17^

Successful weight loss by women has been reported with inclusion of some form of an interactive approach, such as group sessions designed in accordance with social cognitive theory,^19^ including programs designed and led by registered dietitians^20, 21, 22^ in accordance with guidelines from the Academy of Nutrition and Dietetics.^23^ Black women, however, have generally been found to achieve less weight loss than white women in behavioral weight loss interventions.^24^ Alternatively, internet-based educational tools have also been successful in achieving weight loss,^12, 18, 25, 26, 27^ although efficacy in underrepresented minorities is unknown. Therefore, the objectives of this study were to: (i) determine the added weight and fat mass loss efficacy of small-group sessions led by a registered dietitian to a work site wellness program that provided internet-based nutrition and lifestyle information, and access to exercise facilities close to the work site in a randomized clinical trial and (ii) examine racial (black and white) differences in response to the study intervention. We reasoned that small-group nutrition education—designed and conducted by registered dietitians—would be an effective method for weight loss in a racially diverse population of overweight female employees when combined with internet-based wellness information and exercise resources that could be accessed by participants at their convenience.

## Materials and methods

### Study population

Overweight (body mass index (BMI) 25–<30 kg m^−2^) and obese (BMI ⩾30 kg m^−2^) nondiabetic (fasting glucose <126 mg dl^−1^) female employees of the National Institutes of Health, Bethesda, MD, USA, who self-identified as healthy and not active participants in exercise or weight-reduction programs were recruited by campus announcements. Women were excluded from the study for anemia (hemoglobin <11 g dl^−1^) regardless of etiology; liver, kidney or thyroid disease by screening blood work; or recent (<2 months) initiation of prescription medication. Prescription medications at stable doses for at least 2 months—including hormonal preparations for thyroid dysfunction, birth control or post-menopausal symptoms— were permitted, but change in medications during the study was pre-specified as an exclusion criterion from further participation because of possible effects on study end points. The protocol was approved by the Institutional Review Board of the National Heart, Lung, and Blood Institute, and registered in ClinicalTrials.gov (NCT00666172) before study initiation. All subjects provided informed written consent. Race and ethnicity were identified by self-report. All testing was performed after an overnight fast, and for premenopausal women, within the first 10 days of their menstrual cycle. Subjects were compensated for time and inconvenience of testing but were otherwise not paid for participation.

### Weight loss and fitness resources

Each participant was provided internet-based wellness information created by the NHLBI for employees (recent version can be found at http://apps.nhlbi.nih.gov/keepthebeat) that included recommendations from the Department of Health and Human Services and the US Department of Agriculture.^28^ The website included walking paths around the NIH Bethesda campus, sample menus, healthful lifestyle information and tools for counting calories. Each participant was given a pedometer (Walk4Life, Plainfield, IL, USA) with instructions to increase average daily step count by 5000 steps over their baseline average, and given card–key access to private fitness rooms located in three buildings on campus, each equipped with aerobic exercise equipment (for example, treadmill, elliptical machine and supine bicycle). Participants were also encouraged to continue physical activity—especially walking—on days when not at work.

### Study groups

Subjects were randomized by the Clinical Center Pharmacy Development Service into either internet wellness information combined with nutrition education group sessions (WI+GS) or internet wellness information alone (WI) using a table of random numbers with block size not revealed to the investigators. Those randomized to WI+GS were instructed to attend nutrition education sessions (weekly for the first 3 months, monthly for the last 3 months) in small groups (⩽10 persons per session) conducted by a registered dietitian; attendance was recorded. Entry into classes was on a rolling basis depending on when the subject enrolled in the study; all subjects received the same information over the course of 6 months participation. Sessions were held at noon and participants were encouraged to bring lunch so that there was minimal disruption from their work responsibilities. Before the initial group session, subjects met privately with the dietitian to set weight loss goals for the 6-month program. After reviewing their 3-day food record, the dietitian provided a handout delineating their calculated energy needs to promote weight loss, along with a daily meal plan reflecting this individualized calorie level. The dietitians encouraged class participants to keep daily food journals throughout the study as a technique to improve dietary compliance. The first 3 months of the group education sessions consisted of a 12-week curriculum that was created by three registered dietitians associated with this study (NGS, JdJ and BDF) based in part on NHLBI's Aim for a Healthy Weight Program (most recent version can be found at http://www.nhlbi.nih.gov/resources/obesity/education/aim.htm). The curriculum (Table 1) provided pertinent healthful information that could easily be incorporated into the women's lifestyle. Each 45 min session included an individual (private) weigh-in, homework questions related to previous sessions to be discussed by attendees and a presentation on nutrition information relevant to weight management and cardiovascular health. After 3 months, WI+GS participants transitioned to monthly classes for the remaining 3 months, which also included weigh-ins and were conducted by a dietitian, with focus on weight loss maintenance.

### Testing

All testing was performed at the NIH Clinical Center, Bethesda, MD, USA. Baseline, 3 month and 6-month visits included weight measurement; baseline and 6-month visits included waist (at the uppermost border of the iliac crest) and hip circumference (at the maximum protuberance of the buttocks) measurements to the nearest millimeter with a non-stretch tension tape measure (Gulick II, Gays Mills, WI, USA), fasting blood work (glucose, insulin and lipids), and total fat mass and percent truncal fat measured by dual-energy X-ray absorptiometry (DXA; iDXA Software Encore 11.10, GE Lunar Medical Systems, Madison, WI, USA). Exercise performance was measured at baseline and 5 months during graded treadmill exercise (standard Bruce protocol^29^) with a SensorMedics Vmax Spectra 229c metabolic cart (CareFusion, San Diego, CA, USA) for the analysis of oxygen consumption at peak exercise (peak VO_2_). Insulin sensitivity was assessed by the homeostasis model assessment for insulin resistance (HOMA-IR).^30^ Women were asked to maintain a diary of daily pedometer counts throughout the 6-month study. All testing was conducted and analyzed by investigators who were blinded to randomized assignment.

### Statistical analysis

The primary end points of this study were the differences in weight reduction between the WI+GS and WI groups from baseline to 3 months and to 6 months follow-up, with analysis of differences in weight loss by race (black, white). We performed an intention-to-treat analysis in which weight loss for subjects who withdrew from the study after at least the 3-month weight measurement was imputed on the basis of 0.3 kg per month regained weight^31^ as well as weight loss analysis for subjects who completed the 6-month program. Participants who withdrew from the study before 3 months were excluded from analysis because no end point data were available. Secondary outcomes included: changes in total fat mass, truncal fat (as percent of total fat), abdominal and hip circumferences, lipid levels, insulin sensitivity and exercise performance from baseline to 6 months for participants who completed the entire program. *T*-tests or Mann–Whitney–Wilcoxon test for continuous data and *χ*^2^ proportionality test were performed using InStat biostatistics software (GraphPad Software, Version 3.06, 2003; San Diego, CA, USA). Associations between frequency of attendance at group sessions and weight or fat mass loss were performed using either Pearson's correlation or Spearman's rank correlation, as appropriate. In addition, separate repeated-measures mixed-effects models were fit to the primary outcome of weight change at 3 and 6 months, and each of the secondary outcomes at 6 months. Adjusted models were evaluated to determine the treatment (WI+GS or WI) effect, time effect and the treatment × time interaction effect. All models were adjusted for age, race and baseline BMI. Skewed data were log-transformed. Differences in treatment effect within racial groups were assessed with a race × treatment interaction term (limited to non-Hispanic black and white women, given the small number of other racial groups in the study population). All adjusted model analyses were performed using the SAS statistical analysis package (SAS User's Guide: Statistics, Version 9 Edition: SAS Institute Inc, Cary, NC, USA). A *P*-value ⩽0.05 was considered statistically significant. The study had 80% power at a two-sided type I error of 0.05 to detect a difference in weight change between WI+GS and WI groups of 2 kg, assuming a population s.d. of 3 kg and a withdrawal rate of 30%. Data are reported as mean±s.d.

## Results

### Study participants

**Figure 1:**
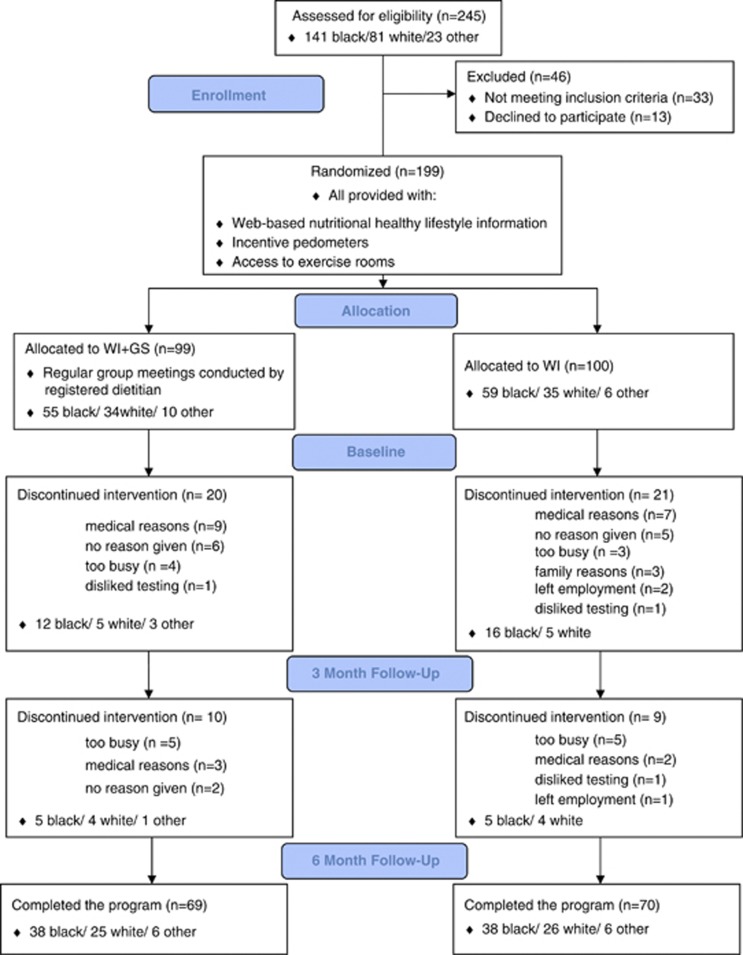
Flow chart of participants in a protocol randomizing participants to internet-based wellness information and nutrition education group sessions (WI+GS) vs wellness information only (WI).

After screening 245 nondiabetic women who responded to the initial advertisement, 199 were consented to the protocol (Figure 1). BMI for the group averaged 33.9±6.3 kg m^−2^, ranged 25.0–57.7 kg m^−2^. Fifteen subjects reported taking HMG-CoA reductase inhibitor (statin) therapy for hypercholesterolemia, with stable drug preparation and dose for at least 2 months before study entry. There were no significant differences in demographics or clinical characteristics between WI+GS and WI groups at baseline (Table 2).

### Program participation

One hundred fifty-eight participants completed at least 3 months of participation, with 139 women completing the entire 6-month program (Figure 1). Sixty women withdrew from the study before completing 6 months participation (30 subjects in each group), with reasons for discontinuation of participation indicated in the flow chart.

### Study outcomes

**Figure 2:**
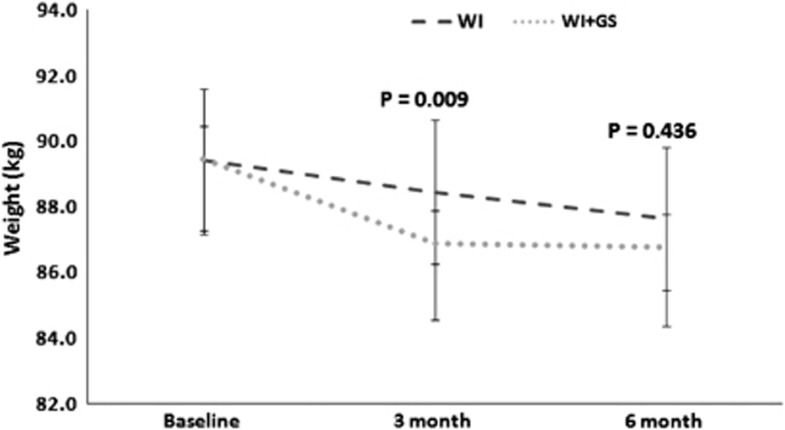
Weight loss for the 158 women (79 receiving internet wellness information combined with nutrition education group sessions (WI+GS), 79 receiving internet wellness information alone (WI)) who participated in the protocol for at least 3 months. Repeated-measures mixed-effects models (adjusted for age, race and baseline BMI) were used to assess the effect of 3 and 6 months participation and WI+GS vs WI assignment on weight loss.

Repeated-measures mixed models for weight change (adjusted for age, race and baseline BMI) for the 158 women (79 in WI+GS and 79 in WI) who remained in the protocol at 3 months showed a statistically significant time effect for weight loss (*P*<0.001) and treatment × time interaction (*P*=0.009), with greater weight loss for the 79 participants in the WI+GS compared with the 79 in WI (−2.2±2.8 vs −1.0±3.0 kg) (Figure 2). By intention-to-treat analysis, at 6 months a statistically significant time effect was evident for weight loss (*P*<0.001), but the treatment × time interaction was not significant (*P*=0.436). In all, 21 of 79 (27%) women in the WI+GS group achieved ⩾5% weight loss from baseline, as did 14 of 79 (18%) women in the WI group (*P*=0.180). The average pedometer counts were similar between the WI+GS subjects and the WI subjects (6239±3393 vs 7110±3325 steps per day, *P*=0.136).

Changes in weight, adiposity, exercise performance, insulin sensitivity and lipids for the 139 women who completed the 6-month program are reported in Table 3. The time effect for measures of adiposity, exercise performance, insulin sensitivity and blood lipids was likewise statistically significant (all *P*<0.05). The treatment × time interaction, however, was not statistically significant for these secondary outcomes (all *P*>0.05).

### Attendance at group sessions and weight loss

For WI+GS subjects, frequency of attendance at the sessions ranged from 0% (two subjects) to 100% (three subjects) with a median attendance frequency of 73% of sessions. Attendance at group sessions was inversely associated with changes in weight (*r*=−0.368, *P*=0.002) and fat mass (*r*=−0.310, *P*=0.011), such that those who attended group sessions most frequently achieved the greatest weight and fat mass loss. Attendance at group meetings by WI+GS subjects was independent of age (*P*=0.429) and race (*P*=0.242).

### Study outcome by race

Compared with white women, black women were significantly younger (45±9 vs 48±11 years; *P*=0.031), more obese (BMI 35.2±6.4 vs 31.7±5.3 kg m^−2^; *P*<0.001) and had lower peak VO_2_ (21.7±4.7 vs 26.0±4.8 ml O_2_ kg^−1^ min^−1^; *P*<0.001) at the start of the study. Similar proportions of blacks and whites completed the study (67% vs 74%, *P*=0.303). Average pedometer counts trended to be higher in white subjects compared with black subjects (7301±3195 vs 6162±3422 steps per day, *P*=0.061). Time effect for weight loss at 3 and 6 months was significant for black women and for white women (*P*<0.001). There was no evidence of a differential effect of the WI+GS vs WI on weight loss for black vs white women in the study based on intention-to-treat analysis, as the race × treatment interaction was not statistically significant for the primary outcome of weight loss at 3 months (*P*=0.816) or at 6 months (*P*=0.819). For those black and white women who completed the 6-month program, the time effect for measures of adiposity, exercise performance, insulin sensitivity and blood lipids was statistically significant for both races (all *P*<0.05). The race × treatment interaction, however, was not statistically significant for weight and fat mass loss and for all secondary outcomes, and there were no statistically significant differences in improvement in outcomes by race (Table 4). No statistically significant difference between blacks and whites was observed in the proportion of women achieving ⩾5% loss of initial weight (21% vs 32%, *P*=0.189), irrespective of group assignment.

## Discussion

In an effort to improve health of employees and reduce costs of disease and absence from work, workplace-based approaches to improve diet and physical activity, ranging from provision of information alone to initiation of exercise programs, and nutrition and behavioral counseling, have been endorsed by the Task Force on Community Preventive Services of the Centers for Disease Control and Prevention, and the American Heart Association.^32^ Consistent with these recommendations, NHLBI created a website (http://apps.nhlbi.nih.gov/keepthebeat) with nutrition and healthy lifestyle information for employees and provided access to exercise rooms on campus to be used before or after work hours, or during breaks through the day. The purpose of our study was to examine the added effect of nutrition education in group sessions—designed and conducted by registered dietitians during the work day—to the provision of internet-based wellness information and exercise resources convenient to the work site on weight loss and other health measures in a racially diverse population of overweight female employees. At 3 months of participation, during which time WI+GS subjects were instructed to attend weekly weigh-in and nutrition education group sessions, subjects in the WI+GS lost significantly more weight from baseline than those randomized to WI alone. By 6 months of participation, both groups had a significant decrease in weight and fat mass and improved cardiovascular risk markers including insulin sensitivity and lipid profile in addition to exercise performance in comparison to baseline measures, but without significant difference between WI+GS and WI groups. Black women achieved similar weight and fat mass reduction, and improvement in exercise performance, insulin sensitivity and lipid profile as did white women. There were no significant differences, however, in the magnitude of changes in outcome measures between women randomized to nutrition education group sessions compared with those who were not. Although the magnitude of weight and fat mass loss for the groups was modest, approximately one-fifth of the WI+GS and WI groups lost over 5% of their initial weight, generally considered to be associated with significant reduction in health risks,^32^ with similar achievement by black and by white women. Weight loss for blacks and for whites in our study was similar to previous reports from multicenter and single center clinical trials (recently reviewed by Fitzgibbon *et al.*^24^) that included women from both races^33, 34^ or black women only,^35, 36, 37, 38, 39, 40, 41, 42^ although less than Diabetes Prevention Program^43^ and Weight Loss Management Trial^44^ that included women at higher risk for cardiovascular disease. Our study extends these findings to the work site with provision of information, group sessions and exercise resources made convenient to the employee.

A potential explanation for failure of the nutrition education group sessions to achieve greater weight loss and improvement in secondary outcomes compared with controls at 6 months is that all participants were provided internet-based information created by NHLBI on nutrition, lifestyle choices and access to convenient exercise resources, which together may have masked contribution from nutrition education sessions. However, WI+GS participants did achieve significantly greater weight loss compared with the WI group at 3 months, during which the emphasis in the instructional sessions was on weight loss. Weight loss stabilized with 3 additional months of participation, during which WI+GS transitioned from weekly to monthly group sessions with instructional emphasis shifted to weight loss maintenance. By contrast, the WI group progressively lost weight during this interval and closely approximated weight loss achieved by the WI+GS subjects at 6 months. This raises the possibility that the weekly weigh-ins and nutrition education sessions focusing on weight loss for the initial 3 months were of value to the WI+GS group, and that a move to monthly sessions and shift in instructional focus for the remaining 3 months was premature for many subjects. Accordingly, some participants might have benefitted from continuation of weekly instruction to facilitate further weight loss, consistent with programs that focused special attention on those unable to achieve weight loss goals. Thus, Jakicic *et al.*^45^ tested a stepped-care model in a randomized trial of 363 overweight and obese adults (83 female, 33% nonwhite) that included increased contact frequency (group sessions, mail, telephone and individual sessions) and even meal replacements for participants missing weight loss goals at 3-month intervals. This intervention approach resulted in ∼7% weight loss after 18 months of participation. Finally, our study may have been underpowered to show a smaller difference in outcomes favoring the use of nutrition education sessions, at least as designed for our study.

Adherence to the program was a major concern for our study, as true for previously reported weight loss trials, with several reporting a lower retention rate for blacks than whites.^24^ Thirty percent of women who responded to the initial program announcement posted at our institution, were found to be eligible, consented to the protocol and began the program, did not complete 6 months of participation. The most common explanation for dropping out was work commitments that did not allow time for exercise in the facilities provided to them or attendance at the nutrition education group sessions for those randomized to this intervention. Solutions to this may include not only greater appreciation for the health benefits on the part of supervisors for employees, but also improved time management to allow breaks for exercise during the work day, if not before or following work. Interactive web-based provision of information and self-monitoring of weight loss as well as email reminders to promote continued engagement in weight loss programs, as used in the POWER study,^25^ might have achieved greater engagement with our weight loss program.

We considered whether nutritional instruction from the registered dietitians in our study, who were all white, may not have been of value to the majority of participants who were black. In this regard, Batch *et al.*^46^ found that race concordance between behavioral interventionist leader and participants in Weight Loss Maintenance trial was not associated with weight loss for black participants. There was no significant difference, however, in the number of blacks randomized to the weekly nutrition education sessions who dropped out of the study in comparison with whites. In addition, there was no significant difference in weight loss between blacks and whites randomized to this intervention. When analyzed on the basis of attendance, which was similar for black women and for white women, WI+GS subjects who were frequent attendees at the weigh-in and educational sessions achieved greater weight and fat mass loss than those randomized to the WI+GS who attended infrequently. The relevance of attendance in our study is consistent with findings of the POUNDS LOST study, in which attendance at group counseling sessions was predictive of weight loss at 2 years.^31^ Personal intervention in the form of group sessions may also be useful in maintenance of weight loss. In this regard, the Weight Loss Maintenance Collaborative Research Group found that monthly personal contact with the specialist who had previously led 20 weekly group sessions resulted in less weight regain than use of an interactive website.^47^

Strengths of our study include the large and racially diverse composition of the study population, and the association of weight and fat mass loss both in black women and in white women with improvement in health measures including insulin sensitivity and lipid profile. In addition, exercise rooms and equipment were provided at the workplace to all study participants for their convenience, which may have facilitated similar improvement in exercise performance for black and for white participants. A limitation of the study is the possibility that continuation of weekly nutrition education sessions might have facilitated continued weight loss in the WI+GS group, whose weight appeared to stabilize following transition from weekly to monthly sessions with instructional emphasis shifted to weight loss maintenance. Further, the duration of the study was 6 months: Continuation for a longer period, even with monthly sessions, might have favored the nutrition education group intervention regarding continued weight loss or maintenance of weight loss. Others have shown that continuation of group sessions may sustain weight loss over time.^47^ Additional limitations of this study include the possibility that greater weight loss may have been achievable by use of portable devices to self-monitor diet and physical activity as reported in the Move! Trial conducted in a Veterans Administration cohort.^48^ In addition, sessions including motivational interviewing, which were not included in our nutrition education sessions, have also been reported to be effective in several weight loss trials.^49^

## Conclusions

Overweight women provided with internet-based wellness information and exercise resources at the work site lost weight and fat mass, with similar achievement by black and white women. Additional weight loss benefit of nutrition education sessions, apparent at 3 months, was lost by 6 months and may require special emphasis on subjects who fail to achieve weight loss goals to show continued value.

## Figures and Tables

**Table 1:** Class topics for nutrition education sessions

*Session # for months 1–3: weekly class topic*
Session 1: energy balance/food journaling
Session 2: what counts as a serving and low calorie menu plans
Session 3: reading labels and smart shopping
Session 4: keeping your portions under control
Session 5: making better choices when you eat out
Session 6: food preparation and food storage
Session 7: action plan development
Session 8: heart health
Session 9: eating cues and mindful eating
Session 10: beverages: think about what you drink
Session 11: eating hints for the workplace
Session 12: restaurant eating

*Session # for months 4–6: monthly class topic*
Session 1: staying motivated
Session 2: tips for success
Session 3: National Weight Control Registry

**Table 2:** Baseline clinical characteristics–internet-based wellness information and nutrition education group sessions (WI+GS) vs wellness information only (WI)

	*WI+GS (*n*=99)*	*WI (*n*=100)*	P*-value*
Age	47±9	45±12	0.191
*Race/ethnicity*			
Black (%)	56	59	0.942
White (%)	33	34	
Asian (%)	4	1	
Hispanic (black or white) (%)	7	6	
			
*Adiposity*			
BMI (kg m^−2^)	34.0±6.2	33.8±6.6	0.748
Weight (kg)	91.3±18.7	90.6±18.3	0.657
Fat mass (kg)	39.9±11.9	40.6±12.5	0.829
Truncal fat (%)	46±6	47±6	0.557
Abdominal circumference (cm)	104.4±14.9	105.7±14.5	0.518
Hip circumference (cm)	118.5±12.6	118.0±12.6	0.646
			
*Exercise performance*			
Duration (s)	406±96	403±107	0.786
Peak VO_2_ (ml kg^−1^ min^−1^)	23.3±5.0	23.4±5.5	0.812
			
*Insulin sensitivity*			
HOMA	2.3±2.1	2.1±1.6	0.852
			
*Lipids (*n*=89, 96)*			
Total cholesterol (mg dl^−1^)	185±33	187±34	0.685
LDL-cholesterol (mg dl^−1^)	114±28	112±31	0.563
HDL-cholesterol (mg dl^−1^)	53±13	56±14	0.17
Triglycerides (mg dl^−1^)	88±46	93±50	0.592

Abbreviations: BMI, body mass index (weight divided by squared height); HDL, high-density lipoprotein; HOMA, homeostasis model assessment; LDL, low-density lipoprotein; VO_2_, oxygen consumption.

Lipid measurements reported for subjects not taking HMG-CoA reductase inhibitor (statin) medications. Data are presented as mean values±s.d. or as frequency (%).

**Table 3:** Baseline to 6-month changes in outcome measures for women who completed 6-month program—internet-based wellness information and nutrition education group sessions (WI+GS) vs wellness information only (WI)

	*WI+GS (*n*=69)*	*WI (*n*=70)*	*Time effect* P*-value*	*Treatment effect* P*-value*	*Treatment* × *time effect* P-*value*
*Adiposity*					
Weight (kg)	−2.7±3.9	−2.0±3.9	<0.001	0.784	0.332
Fat mass (kg)	−2.2±3.1	−1.7±3.7	<0.001	0.148	0.411
Truncal fat (%)	−1.8±2.9	−1.3±3.1	<0.001	0.143	0.423
Abdominal circumference (cm)	−3.4±5.2	−2.7±5.4	<0.001	0.266	0.461
Hip circumference (cm)	−2.4±4.6	−1.1±4.2	<0.001	0.920	0.154
					
*Exercise performance*					
Duration (s)	+47±60	+50±69	<0.001	0.408	0.754
Peak VO_2_ (ml kg^−1^ min^−1^)	+1.7±3.1	+1.5±3.1	<0.001	0.522	0.754
					
*Insulin sensitivity*					
HOMA	−0.5±1.0	−0.1±1.3	<0.001	0.906	0.099
					
*Lipids (*n*=65, 69)*					
Total cholesterol (mg dl^−1^)	−13±25	−8±23	<0.001	0.313	0.252
LDL-cholesterol (mg dl^−1^)	−8±18	−5±23	<0.001	0.978	0.458
HDL-cholesterol (mg dl^−1^)	−2±10	−2±8	0.016	0.034	0.587
Triglycerides (mg dl^−1^)	−10±32	−9±29	<0.001	0.577	0.867

Abbreviations: BMI, body mass index (weight divided by squared height); HDL, high-density lipoprotein; HOMA, homeostasis model assessment; LDL, low-density lipoprotein; VO_2_, oxygen consumption.

Lipid measurements reported for subjects not taking HMG-CoA reductase inhibitor (statin) medications. Data are presented as mean values±s.d.

Repeated-measures mixed-effects models (adjusted for age, race and baseline BMI) were used to assess the effect of 6 months participation (time) and WI+GS vs WI assignment (treatment) on the primary outcome of weight loss and all secondary outcomes.

**Table 4:** Baseline to 6-month changes in outcome measures for black and for white women who completed 6-month program

	*Black (*n*=76)*	*White (*n*=51)*	P*-value*
*Adiposity*			
Weight (kg)	−1.8±3.4	−3.3±5.2	0.255
Fat mass (kg)	−1.6±2.7	−2.5±4.3	0.532
Truncal fat (%)	−1.3±2.5	−1.7±3.7	0.878
Abdominal circumference (cm)	−2.1±4.4	−4.1±6.2	0.066
Hip circumference (cm)	−1.5±3.6	−1.9±5.6	0.909
			
*Exercise performance*			
Duration (s)	+47±67	+50±65	0.688
Peak VO_2_ (ml kg^−1^ min^−1^)	+1.4±3.2	+1.7±3.1	0.585
			
*Insulin sensitivity*			
HOMA	−0.4±1.1	−0.4±1.1	0.947
			
*Lipids (*n*=70, 42)*			
Total cholesterol (mg dl^−1^)	−10±19	−11±31	0.760
LDL-cholesterol (mg dl^−1^)	−6±18	−7±25	0.967
HDL-cholesterol (mg dl^−1^)	−2±8	−3±12	0.946
Triglycerides (mg dl^−1^)	−8±29	−10±32	0.501

Abbreviations: HDL, high-density lipoprotein; HOMA-IR, homeostasis model assessment for insulin resistance; LDL, low-density lipoprotein; VO_2_, oxygen consumption.

Lipid measurements reported for subjects not taking HMG-CoA reductase inhibitor (statin) medications. Data are presented as mean values±s.d.
